# The Effect of HIV-Hepatitis C Co-Infection on Bone Mineral Density and Fracture: A Meta-Analysis

**DOI:** 10.1371/journal.pone.0101493

**Published:** 2014-07-17

**Authors:** Tyler J. O’Neill, Laura Rivera, Vladi Struchkov, Ahmad Zaheen, Hla-Hla Thein

**Affiliations:** 1 Dalla Lana School of Public Health, University of Toronto, Toronto, Ontario, Canada; 2 Ontario Institute for Cancer Research/Cancer Care Ontario, Toronto, Ontario, Canada; 3 Faculty of Medicine, University of Toronto, Toronto, Ontario, Canada; Harvard Medical School, United States of America

## Abstract

**Objective:**

There is a variable body of evidence on adverse bone outcomes in HIV patients co-infected with hepatitis C virus (HCV). We examined the association of HIV/HCV co-infection on osteoporosis or osteopenia (reduced bone mineral density; BMD) and fracture.

**Design:**

Systematic review and random effects meta-analyses.

**Methods:**

A systematic literature search was conducted for articles published in English up to 1 April 2013. All studies reporting either BMD (g/cm^2^, or as a T-score) or incident fractures in HIV/HCV co-infected patients compared to either HIV mono-infected or HIV/HCV uninfected/seronegative controls were included. Random effects meta-analyses estimated the pooled odds ratio (OR) and the relative risk (RR) and associated 95% confidence intervals (CI).

**Results:**

Thirteen eligible publications (BMD N = 6; Fracture = 7) of 2,064 identified were included with a total of 427,352 subjects. No publications reported data on HCV mono-infected controls. Meta-analysis of cross-sectional studies confirmed that low bone mineral density was increasingly prevalent among co-infected patients compared to HIV mono-infected controls (pooled OR 1.98, 95% CI 1.18, 3.31) but not those uninfected (pooled OR 1.47, 95% CI 0.78, 2.78). Significant association between co-infection and fracture was found compared to HIV mono-infected from cohort and case-control studies (pooled RR 1.57, 95% CI 1.33, 1.86) and compared to HIV/HCV uninfected from cohort (pooled RR 2.46, 95% CI 1.03, 3.88) and cross-sectional studies (pooled OR 2.30, 95% CI 2.09, 2.23).

**Conclusions:**

The associations of co-infection with prevalent low BMD and risk of fracture are confirmed in this meta-analysis. Although the mechanisms of HIV/HCV co-infection’s effect on BMD and fracture are not well understood, there is evidence to suggest that adverse outcomes among HIV/HCV co-infected patients are substantial.

## Introduction

The success of antiretroviral therapy (ART) and other advanced therapeutics in reducing the mortality of human immunodeficiency virus (HIV)-infected patients has changed the clinical course of this disease. HIV infected patients are less likely to experience death as a result of ‘AIDS-related’ causes, but are susceptible to ‘non-AIDS-related’ conditions in the course of their treatment [1] including adverse metabolic outcomes of the skeletal system [2], [3]. A common comorbid feature is co-infection with hepatitis C virus (HCV). Studies among HIV patients report hepatic-associated effects of chronic HCV infection, including hepatitis, fibrosis, cirrhosis, and carcinoma, are complicated by HIV co-infection [4]. The effect of non-hepatic outcomes, including mechanisms leading to low bone mineral density (BMD) and fractures associated with hepatic osteodystrophy, remains unclear due to limited evidence of the effects of co-infection [5], [6], [7].

Osteoporosis is a systemic skeletal disorder characterized by low bone mass. Osteopenia (BMD <1 standard deviations of the mean BMD of a sex-matched, young healthy population below normal BMD, i.e. T-score –1 to –2.5) often precedes osteoporosis (i.e. T-score <–2.5), predisposing affected patients to fracture [8], [9], [10]. Low BMD is prevalent in HIV patients [11]. Although the incidence of fractures among HIV patients is low, they can be debilitating and potentially life-threatening leading to lower quality of life [12], [13], [14], [15], [16], [17], [18], [19], [20]. Studies have found higher rates of osteoporotic-associated fractures in HIV patients compared to uninfected controls matched for age [21], [22]. The use of ART by HIV mono-patients has also been implicated in exacerbating the severity of adverse skeletal outcomes [3], [11], [21]. However, it is unclear if it is HIV infection per se, ART, or both that lead to bone loss [10].

Multiple cross-sectional studies have reported an association between chronic HCV infections, reduced BMD [23], [24], [25], [26], and increased risk of fracture [27]. HCV co-infection, compared to HIV mono-infected patients, significantly increases the risk of fractures at many sites [7], [28], [29]. Low BMD is a recognized complication of HIV infection [11], HIV/HCV co-infection [30], or use of ART [3]. Thus, the risk of fracture in co-infected patients may be greater than that of HIV or HCV mono-infected patients. Not only is co-infection globally prevalent [31], with osteoporotic fractures leading to $12–18 billion USD in direct health care costs annually. Loss of productivity from work because of continual pain, increased absenteeism, and psychological factors add significantly to the total cost of low BMD [32].

Conflicting evidence suggests that adverse bone outcomes are common amongst HIV [2] and HCV [29] mono-infected patients. For example, Lo Re et al. [7] did not find any significant difference in risk of hip fracture between HIV/HCV co-infected and HIV mono-infected patients. While in contrast, Lo Re et al. [30] reported a higher rate of hip fracture was found in co-infected patients compared to HCV mono-infected. However, the suspected synergistic effects of co-infection, including increased inflammation and higher risk of vitamin D deficiency [3], on these outcomes have not been reported as a primary outcome. Therefore, the objective of this systematic review and meta-analysis is to estimate the association of HIV/HCV co-infection on adverse skeletal outcomes (low BMD and risk of fracture). Furthermore, this study intends to generate an estimate for the associations of co-infection with both reduced BMD and bone fracture to stimulate investigation in this important area of growing understanding among HIV patients’ comorbidities.

## Methods

### Search Strategy

**Figure 1:**
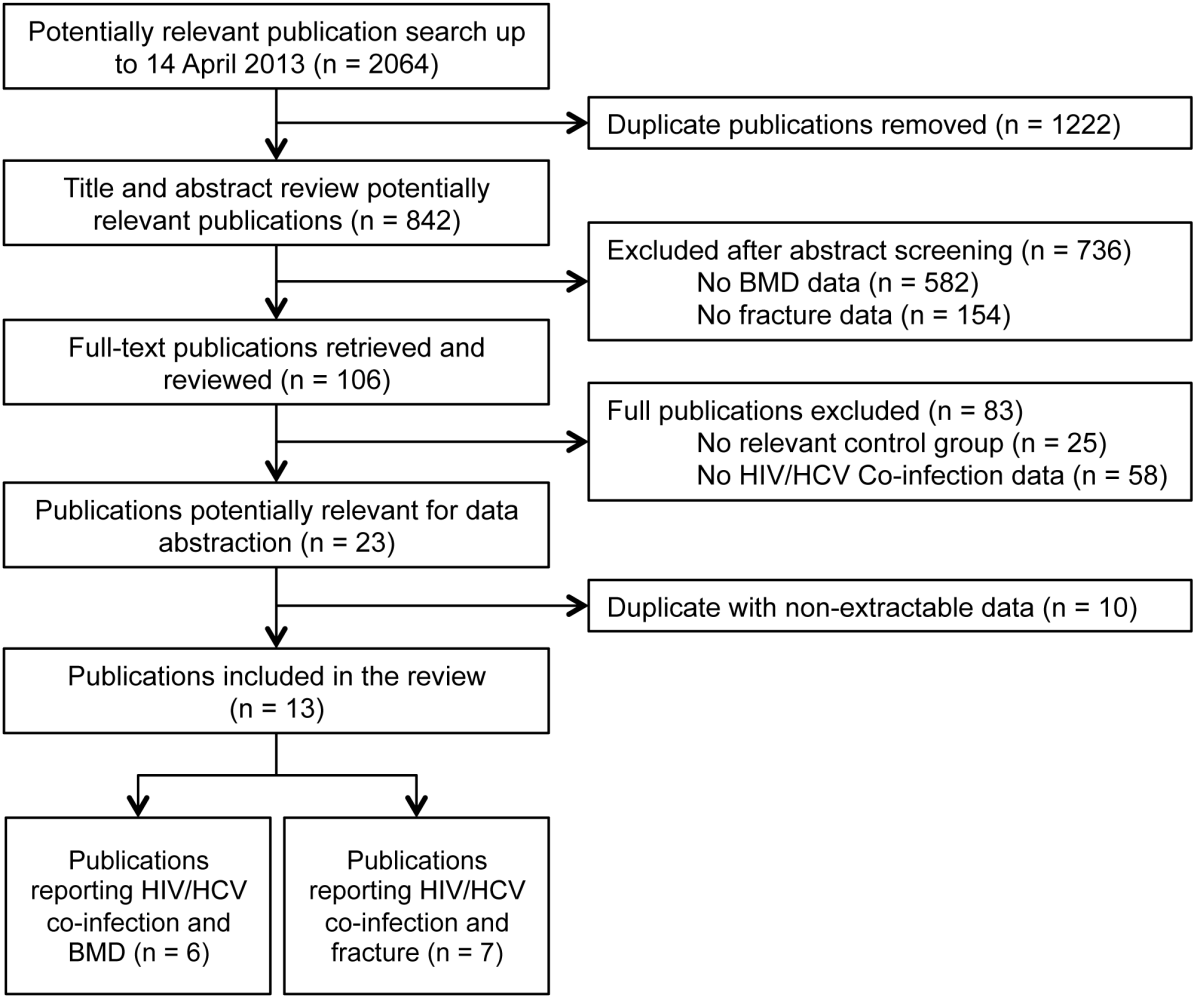
Identification of relevant literature on HIV/HCV co-infection and outcomes of (i) low bone mineral density (BMD) and (ii) fracture. BMD, bone mineral density; HCV, hepatitis C virus; HIV, human immunodeficiency virus.

Using MEDLINE, EMBASE, Cochrane Library (CENTRAL), and Scopus databases available to the University of Toronto, two investigators (VS and LR) performed a systematic search for relevant literature ever published up to 14 April 2013. The search strategy used MeSH terms (“human immunodeficiency virus” OR “human immunodeficiency virus infection” OR “HIV-1”) and (“hepatitis co-infection” OR “hepatitis C” OR “HCV”) and (“bone disease” OR “bone density” OR “bone mineral density” OR “osteoporosis” OR “osteopenia” OR “fracture” OR “bone fracture”). In addition, grey literature (e.g. government reports, theses) was searched in The NLM Gateway, The American Society for Bone and Mineral Research, and The Annual Conference on Retroviruses and Opportunistic Infections. The references of identified publications were crosschecked through review of references of relevant publications included in the review (Figure 1).

### Study Selection

A Publication was considered eligible for inclusion if it: (1) evaluated the association between HIV-1/HCV co-infection and at least one skeletal outcome (BMD or fracture); (2) reported in English; (3) was a full-length, peer-reviewed observational or randomized control trial (RCTs); (4) reported controls that were either HIV or HCV mono-infected, or uninfected/seronegative; (5) had BMD measured by dual energy X-ray absorptiometry (DXA or DEXA); (6) reported the occurrence of osteoporosis or osteopenia as defined by The World Health Organization (WHO) [10]; and/or (7) reported incident pathologic fracture at any location was reported. Pathologic fractures were defined as fractures possibly due to low BMD (at least osteopenia), typically caused by low energy trauma [28]. Studies in non-human populations, individuals <18 years of age, review articles, case reports or studies that lacked relevant controls, studies on HIV-2 or other HIV strains, and those that did not clearly report the outcomes of interest were excluded from the review.

A two-step procedure was used to screen publications by title and abstract followed by full text review by two investigators (LR and AZ) independently. Any discrepancy concerning a publication’s relevancy for inclusion was discussed until consensus was reached. If consensus was not reached, two investigators (HHT and TJO) adjudicated publications.

### Data Abstraction

Using a standardized abstraction form, data were extracted by two reviewers (LR and TJO) applying inclusion and exclusion criteria. Information extracted from each study included: (1) study factors: publication status, year of publication, study design, duration of follow-up, inclusion and exclusion criteria, sample size; (2) host factors: age, gender, body mass index (BMI), baseline osteoporotic risk factors, fracture risk factors; (3) infection factors (HIV or HCV): HIV severity as measured by CD4 lymphocyte count and HIV RNA viral load, duration of HIV or HCV infection, clinical symptoms; (4) HCV treatment and ART duration and protocol(s); and (5) outcome factors, relating to BMD and/or fracture. The WHO (1994) criteria were used to define osteopenia (T-score –1.0 to –2.5) and osteoporosis (T-score <–2.5) at any anatomical site. Normal BMD was defined as a T-score greater than –1.0. Fracture could have occurred at any anatomical site. Ascertainments of fracture from radiological confirmation, reports of diagnosis from medical or insurance records, or by patient self-report were included.

Publications were included if they were population-based and reported co-infection as an independent variable and BMD (g/cm^2^) or incident fracture as outcome measures. Furthermore, included publication were those in which summary estimates, such as odds ratio (OR) or risk ratio (RR) with 95% confidence interval (CI), were reported or if the publication allowed for the estimation of the summary estimates based on reported data.

### Statistical analysis

For case-control and cross-sectional designs, if the adjusted OR of osteopenia or osteoporosis were reported, it was extracted along with covariates controlled for in the model. An unadjusted OR was extracted if an adjusted estimate was not reported. Where necessary, risk was estimated from reported odds according to King and Zeng [33]. The measure of association for cohort studies was summarized using the adjusted RR and 95% CI along with covariates.

Unadjusted measures were estimated where appropriate. For BMD, the mean and its standard deviation (SD) of BMD (g/cm^2^) at any location for cases and controls were extracted. The T-score was then calculated comparing to a healthy 30-year old Caucasian female [10] and unadjusted odds were estimated. The odds of having a T-score consistent with osteopenia or osteoporosis were pooled to estimate a dichotomous outcome of low BMD. For publications reporting fracture data (e.g. number of fractures in each group), odds or risk and exact 95% CI were estimated using the reported number of individuals with incident fractures compared to controls.

Pooled analyses, comparing co-infected patients with mono- or uninfected controls, were estimated using random effects models to account for both within- and between-study variability [34]. An inverse variance method was used to weight each estimate. If BMD was measured with more than one control group (e.g. study reported both HIV negative and mono-infected controls) and an average was not reported, data were reported as separate studies within a single publication. If a study evaluated BMD at multiple time points, the value from the first measurement (most reflective of baseline) was selected to avoid dependence. Similarly, only incident fractures were considered. When sufficient information was provided to stratify data for multiple groups (e.g. gender, age), separate estimates were calculated for each group; again, treating the data as separate studies from a single publication.

The I^2^ statistic was estimated as a measure of the total variation in point estimates attributable to between-study heterogeneity [35], [36]. The magnitude was interpreted as low (I^2^≤25%), medium (I^2^ = 50%) and high heterogeneity (I^2^≥75%). A χ^2^ test was used to assess statistical significance of heterogeneity within each analysis (α = 0.05). Sensitivity analyses were employed to determine the causes of heterogeneity by evaluating whether BMD or fracture risk varied significantly based on demographic differences amongst publications. Furthermore, the included publications were selected for step-wise analysis of heterogeneity. Studies were systematically removed and replaced to estimate individual effects on pooled analyses. We evaluated publication bias using Egger regression asymmetry test and Begg’s t. The analysis was conducted using Review Manager (RevMan, version 5.2, The Cochrane Collaboration, Copenhagen) and STATA (version 12, StataCorp, College Station, Tx). PRISMA reporting guidelines were adhered to in this publication (Checklist S1).

## Results

### Selection of Studies

The systematic search identified 2,064 potentially relevant publications (Figure 1). After exclusion of duplicates, and titles and abstracts were screened, 106 independent full-text publications were further evaluated. Studies were subsequently excluded due to lack of relevant control group (n = 25) or no co-infection data reported (n = 58), leaving 23 studies for data abstraction. Ten publications were excluded from abstraction because they did not report BMD or fractures in a co-infected population compared to controls. A total of 13 publications (n = 472,352 subjects) met all inclusion criteria and reported outcome(s) of interest. Manual review of included publication references did not yield any additional articles.

Only one publication required author contact [19] to clarify an adjusted OR point estimate and associated 95% CI due to a clerical error in the original publication. All reported adjusted measures of association were abstracted except one comparison of co- to mono-infected patients from Anastos et al. [19] that required unadjusted estimation of the OR based on reported data.

### Demographic and Clinical Characteristics of Included Publications

Published between 2005 and 2012, all included publications reported observational study designs: 7 cohort, 5 cross-sectional, and a single matched case-control (Table 1). Publications were conducted in the United States (n = 7) [7], [19], [29], [38], [39], [40], [41] or Italy (n = 3) [30], [37], [42], with single publications reporting data from Iran [44], Denmark [28], and Australia [43]. Only effective sample sizes (those used to calculate the measure(s) of interest) were extracted, and ranged from 25 adults from a national multiyear, multicenter cohort [39] to 462,656 individuals in a retrospective cohort analysis of US Medicaid patients [7]. The majority of individuals included were male except for two publications [19], [40] with an all-female population. Similar distribution of mean or median ages, BMI, and race was found across publications with no significant differences reported in included publications between cases and controls (Table 1). Lo Re et al. [30] stratified outcomes on gender; Lo Re et al. [7], [30] stratified on both age and gender. In the analysis, these were treated as separate studies within each publication.

**Table 1:** Descriptive characteristics of studies meeting inclusion criteria for low BMD and fracture risk amongst HIV/HCV co-infected patients.

Author	Study Design	Country	N,Total	N, Co-infected(HIV+/HCV+)	Control group(s)		Sex(% Male)		Age (years)	
					N, Monoinfected(HIV+)	N, Uninfected		Ethnicity(% HIV+)		Body Mass Index(kg/m^2^, SD)
Anastos2007	Cross-sectional	USA	387	112	152	123	0	Black (61.3)	36.7 (HIV–)	29.9, 6.72 (HIV–)
									42.1 (HIV+ ART−)	28.9, 6.24 (HIV+ ART−)
								Caucasian (19.7)	40.5 (HIV+ non-PI ART+)	26.8, 5.81(HIV+ non-PI ART+)
								Hispanic (18.9)	43.8 (HIV+ PI ART+) (median)	28.4, 6.87(HIV+ PI ART+)
Badie2011	Cross-sectional	Iran	101	61	40	-	79.2	Middle Eastern(100.0)	39.4, 7.7 (HIV+ ART+)	22.7, 3.40 (HIV+ ART+)
									34.9, 7.3 (HIV+ ART−)	22.7, 3.30 (HIV+ ART−)
									36.6, 10.5 (HIV–)(mean, SD)	22.8, 3.40 (HIV–)
Bedimo2012	Cohort	USA	951	485	-	466	98.0	Black (56.0)	46 (median)	Not reported
								Caucasian (18.0)		
								Hispanic (23.0)		
								Other (2.0)		
Collin2009	Cohort	USA	25	13	-	12	77.2	Not reported	36.2 (median)	22.0 (Not reported)(HIV+)
El-Maouche2011	Cross-sectional	USA	338	Not reported	Not reported	Not reported	55.0	Black (93.8)	42.5, 8 (mean, SD)	23.9, 2.90 (HIV+)*
Fausto2006	Cross-sectional	Italy	161	55	-	106	64.0	Caucasian (100.0)	38.6, 4.18 (mean, SD)	22.9, 3.16 (HIV+ ART−)
										23.1, 3.47 (HIV+ ART+)
Hansen2012	Cohort	Denmark	5306	851	Not reported	-	76.0	Caucasian (80.0)	36.7, 30.5–44.5(mean, 95% CI)	Not reported
Lo Re2009	Cross-sectional	Italy	1237	625	-	612	62.0	Not reported	43, 10–18 (median, IQR)	23.5, 3.44 (HIV+)*
										23.1, 4.10 (HIV+ HCV+)*
Lo Re2012	Cohort	USA	462656		366829	95827	63.0	Black (43.1)	39, 33–46 (mean, IQR)	Not reported
								Caucasian (27.4)		
								Hispanic (8.3)		
								Other (21.1)		
Li Vecchi2012	Cohort	Italy	188	41	76	71	55.9	Caucasian (100.0)	47, 9.7 (HIV+)	22.9, 3.62 (HIV+)*
									49, 49, 11.3 (HIV–)(mean, SD)	23.4, 3.81 (HIV–)*
Yin2010	Cohort	USA	1101	438	663	-	0	Black (56.3)	40.4, 8.8 (HIV+)	28.5, 7.50 (HIV+)
								Caucasian (13.3)		
								Hispanic (27.7)	36.1, 9.9 (HIV–)(mean, SD)	30.0, 8.20 (HIV–)
								Other (3.2)		
Yong2011	Case-control	Australia	46	16	-	30	88.5	Black (3.0)	49.8 (Case)	24.2, 2.91 (Case)*
								Caucasian (92.0)	49.5 (Control) (mean)	25.6, 3.47 (Control)*
								Asian (5.0)		
Young2011	Cohort	USA	193	51	-	142	79.0	Black (33.0)	40, 34–46 (median, IQR)	26.7, 4.83 (HIV+)
								Caucasian (51.8)		
								Hispanic (11.7)		
								Other (3.5)		

HIV, Human Immunodeficiency Virus; HCV, Hepatitis C Virus; ART, antiretroviral therapy; CI, confidence interval; IQR, interquartile range; PI, protease inhibitor; SD, standard deviation.

*Mean (SD) estimated from descriptive categorical data reported in publication.

All studies reported control groups of either HIV mono- [7], [19], [29], [30], [39], [41], [42], [43] or uninfected populations [7], [19], [28], [40], [44]. Lo Re et al. [7] did not find any significant difference in risk of hip fracture between HIV/HCV co-infected and HIV mono-infected patients. Thus, no data were reported in the publication and were not included in the analysis. In a single publication [30] evaluating rates of incident fracture among co-infected patients and HCV mono-infected controls, a higher rate of hip fracture was found in co-infected patients (HR 1.38, 95% CI 1.25, 1.53). In this study, there were insufficient data for pooled estimates using HCV mono-infection as a comparator.

Details of control selection were reported in all publications selected for pooled analyses. Five studies selected controls from within established cohorts: Lo Re et al. [7], [30] used HIV mono- or uninfected controls from the US Medicaid recipients; the ANRS CO8 APROCOC-COPILOTE cohort [39] included HIV mono-infected controls on combination PI-ART; Bedimo et al. [29] used HIV mono-infected patients in the Veterans’ Health Administration Clinical Case Registry data; and Anastos et al. [19] and Yin et al. [40] had HIV mono-infected (HAART naïve, non-PI HAART, PI HAART groups) and uninfected female controls, respectively, both from the Women’s Interagency HIV Study. ART amongst co-infected patients was also reported in Lo Re et al. [7] compared to antiretroviral treated HIV mono-infected patients. Four publications used population-based controls. The NHANES III cohort in Fausto et al. [42] and Badie et al. [44], described hospital-based controls, matched on age, gender, HBV/HCV infection status, and injecting drug use. The NHAMCS-OPDs cohort in Young et al. [41] obtained controls matched on age and gender. Hansen et al. [28] linked the Danish HIV Cohort study to the Danish Civil Registration System and Danish National Hospital Registry. Hospital-based cohorts were included in three publications with age, gender, and duration of HIV infection individually matched by Yong et al. [43] to mono-infected controls, and HIV mono-infected controls at a single hospital included in Lo Re et al. [7], [30].

**Figure 2:**
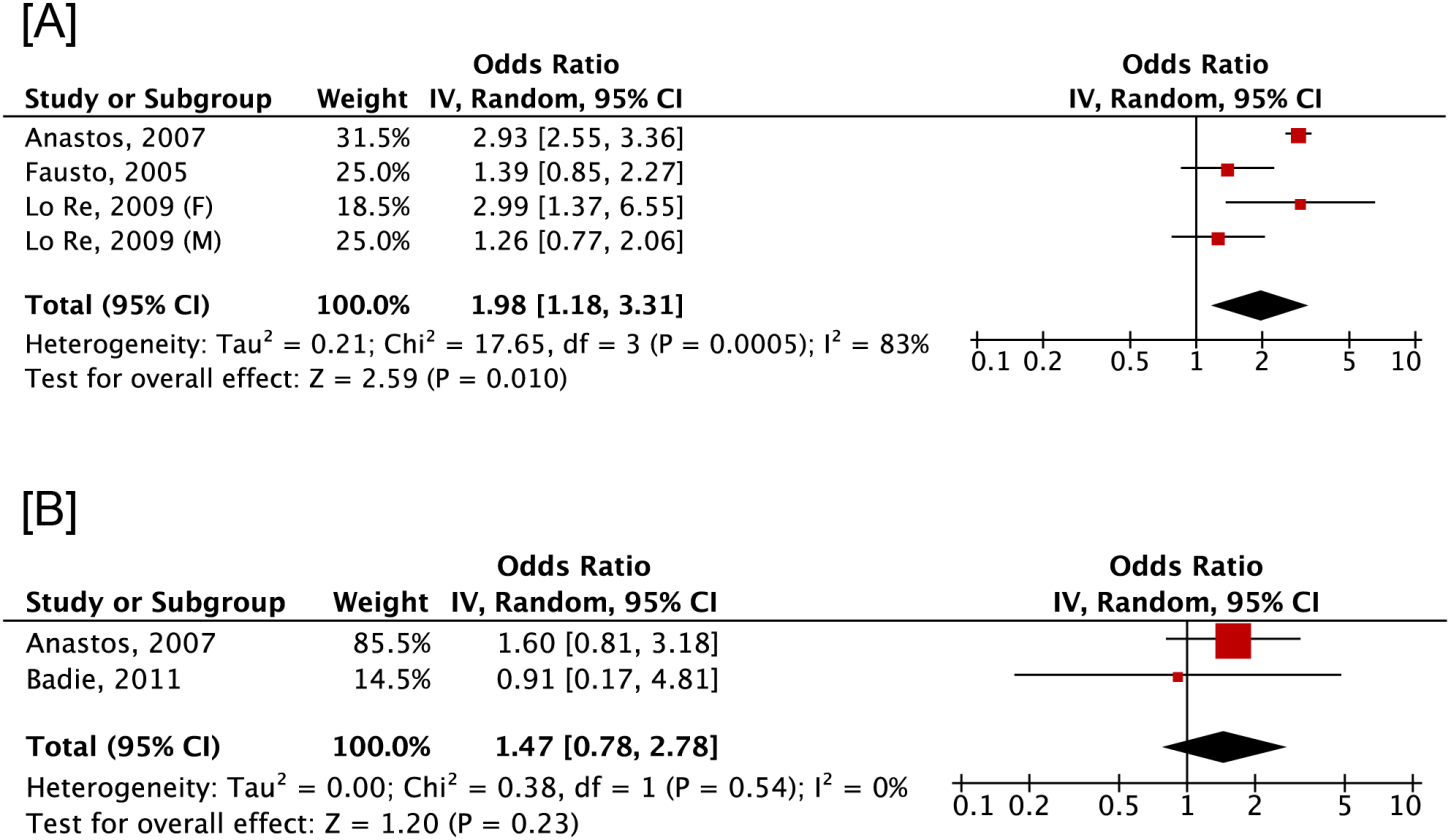
Odds of low bone mineral density between individuals co-infected with HIV and hepatitis C virus compared to HIV mono- (A) or uninfected (B) individuals. HIV, human immunodeficiency virus; HCV, hepatitis C virus.

Individuals on ART at the time of assessment were reported in all publications but one [38] (Table 2). Six publications had an outcome of BMD [19], [30], [37], [38], [42], [44] whereas 8 reported fracture risk [7], [28], [29], [30], [39], [40], [41], [43]. The wrist, hip, and lumbar spine were common locations for assessment of outcomes. All publications used DXA to evaluate BMD. T-scores and Z-scores equivalent to osteopenia or osteoporosis were reported by three [19], [42], [44] and one [30] publications, respectively. In contrast, less than half of the publications identified incident fracture events by means of International Classification of Disease (ICD) version 9 (ICD-9) [29], [43] or 10 (ICD-10) [28]. Diagnosis of fracture was included in the analysis of residents in multiple US states receiving Medicaid [7], [30]. Cohort members from the HIV Outpatient Study (HOPS) [43] treated at non-HOPS sites self-reported incident fracture as did Yin et al. [40] and Young et al. [41], who abstracted incident fractures from patient charts similar to Collin et al. [39]. In the meta-analysis of 4 cross-sectional studies, low BMD was increasingly prevalent among co-infected patients compared to those HIV mono-infected (pooled OR 1.98, 95% CI 1.18, 3.31) despite substantial and significant heterogeneity (I^2^ = 83%, *p*<0.001) (Figure 2). In contrast, no significant changes in BMD odds or heterogeneity were found by pooling data from two cross-sectional that compared co-infected to uninfected individuals (pooled OR 1.47, 95% CI 0.78, 2.78) (I^2^ = 0%, *p* = 0.54). All publications reported adjusted measures.

**Table 2:** Clinical characteristics and reported outcomes of publications included in the HIV/HCV co-infection review.

Author	ART exposure(%)	Reported outcome(s)(classification method)	Location	Co-variates adjusted
Anastos 2007	46.5	BMD (DXA)	Lumbar spine	White, Race, Nadir BMI, HIV+ ART naïve, HIV+ non-PI ART,HIV+ PI ART, post-menopausal
			Femoral neck	
Badie 2011	30.0	BMD (DXA)	Hip	Age, Gender, BMI, Smoking, Alcohol, Exercise, HBV infection,IV drug use, Prison
			Lumbar spine	
Bedimo 2012	69.4	Incident osteoporotic fracture(ICD-9 codes)	Wrist	ART+, CKD, Race, Age, Tobacco use, Diabetes, BMI
			Vertebra	
			Hip	
Collin 2009	100.0	Incident non-stress fracture(patient charts)	Any location	Age, HIV+, HIV-RNA, BMI, Location of birth, PI used first, Alcohol,CD4+ count,
El-Maouche2011	Notreported	BMD (DXA)	Hip	Gender, Age, BMI, Race, Smoking, Alcohol, IV drug use,Hypogonadal/menopausal, HIV+, Methadone use, ART+,Hormone exposure, Vitamin D
			Femoral neck	
			Lumbar spine	
Fausto 2006	70.2	BMD (DXA)	Hip	Gender, Age, CDC Stage, IV drug use, Lypodistrophy, BMI,CD4+ count, HIV–RNA, HAART+, Length of HIV infection,Bone resorption, Bone formation, Vitamin D
			Lumbar spine	
Hansen 2012	78.0	Incident low energy fracture(ICD-10 codes)	Any location	
Lo Re 2009	79.0	BMD (DXA)	Lumbar spine	Age, Gender, BMI, Length of HIV infection, CD4+ count, ART+,Smoking, Alcohol, Exercise, Amenorrhea, eGFR
			Femoral neck	
Lo Re 2012	100	Incident low energy fracture(Medicaid claim codes)	Hip	Age, Gender, State (location), Propensity score
		BMD (DXA)		
Li Vecchi2012	93.0	BMD (DXA)	Lumbar spine	Age, Yogurt intake, CD4+, Drug addiction
			Femoral neck	
Yin 2010	65.6	Incident low energy fracture(self-reported)	Hip	HIV+, Age, Race, BMI, Post-menopausal, Fracture before index, Serum creatinine
			Wrist	
			Spine	
Yong 2011	83.6	Incident non-stress fracture(ICD-9 codes)	Any location	HBV status, Previous opportunistic infection, CD4+ count, BMI,HIV-RNA, Duration of viral suppression, Type of DXA performed
Young 2011	72.7	Incident low energy fracture(patient charts; self-reported)	Wrist	Gender, Age, CD4+ count,
			Vertebra	
			Femoral neck	

ART, antiretroviral therapy; BMI, body mass index; CKD, chronic kidney disease; DXA, Dual energy x-ray absorbiometry; eGFR, estimated glomerular filtration rate; HAART, highly active antiretroviral therapy; HBV, hepatitis B virus; ICD, International Classification of Diseases; PI, protease inhibitor.

**Figure 3:**
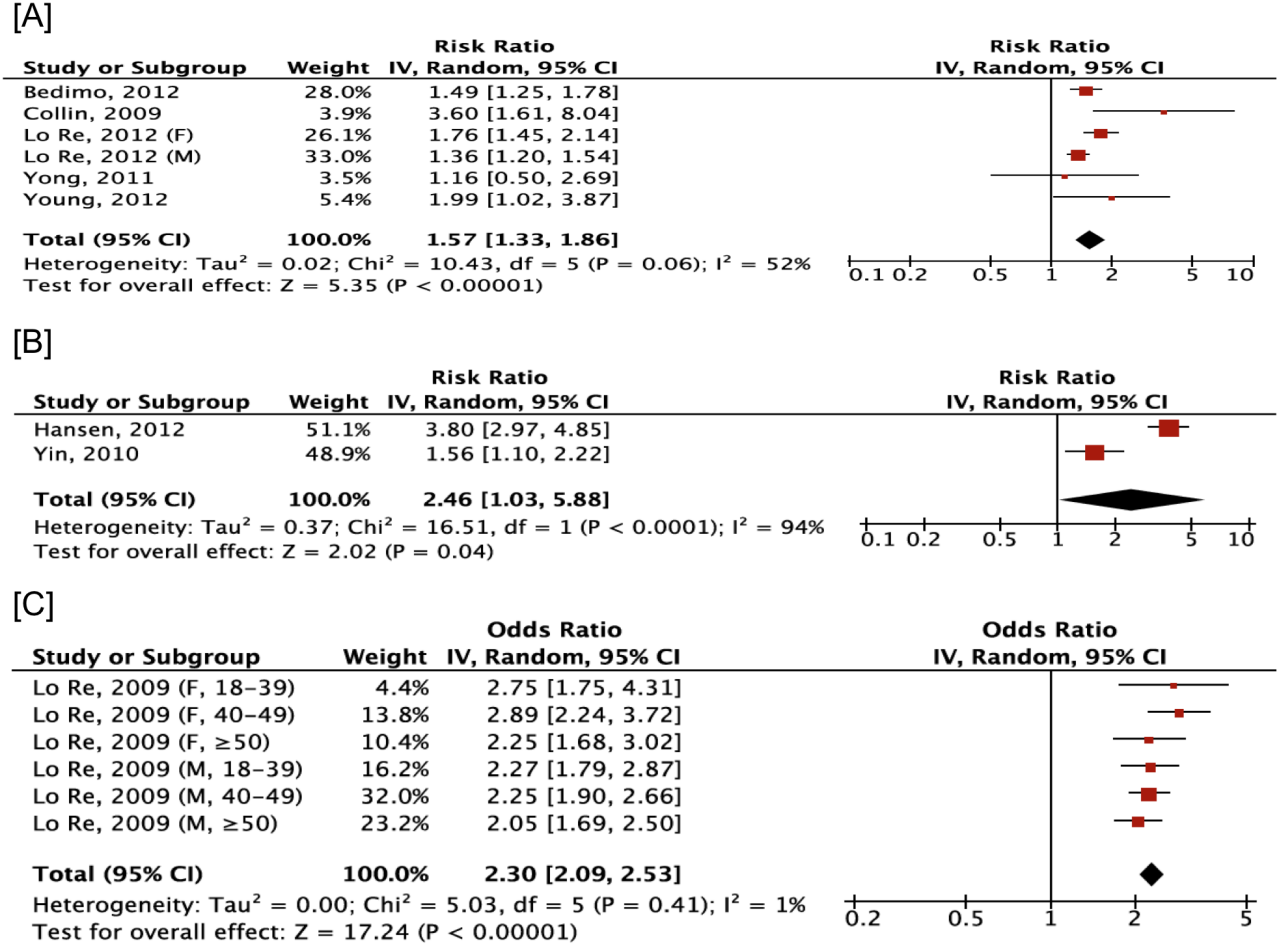
Risk of fracture between individuals co-infected with HIV and hepatitis C virus compared to HIV mono- (A) or uninfected individuals (B), with odds of fracture estimated from cross-sectional studies comparing HIV/HCV co-infected patients to HIV or HCV uninfected individuals (C). HIV, human immunodeficiency virus; HCV, hepatitis C virus.

In the meta-analysis of 5 cohort and 1 case-control study, significant associations between co-infected patients and fracture was estimated compared to HIV mono-infected patients (pooled RR 1.57, 95% CI 1.33, 1.86) with moderate, non-significant heterogeneity (I^2^ = 52%, *p* = 0.06) (Figure 3). An increased association with fracture among co-infected individuals compared to those uninfected with HIV or HCV was found from both cohorts (pooled RR 2.46, 95% CI 1.03, 3.88), despite substantial and significant heterogeneity (I^2^ = 94%, *p*<0.001), and cross-sectional studies (pooled OR 2.3, 95% CI 2.09, 2.53) with no heterogeneity identified (I^2^ = 1.0%, *p* = 0.41). All publications but one [28] reported adjusted measures. There was no evidence of publication bias as indicated by a non-significant Egger test (all *p*>0.05) and Begg’s test (all *p*>0.05) in all analyses.

### Sensitivity analysis

Moderately attenuated heterogeneity and odds of low BMD (co-infected compared to HIV mono-infected) were found using step-wise publication of a single publication [19] (OR 1.58, 95% CI 1.02, 2.45; I^2^ = 44%, *p* = 0.17). Risk of fracture and heterogeneity were substantially attenuated using step-wise publication exclusion of Collin et al. [39] (co-infected compared to HIV mono-infected) (RR 1.51, 95% CI 1.22, 1.71; I^2^ = 31%, *p* = 0.22). No significant changes were observed when either analyses were repeated with omission of the total female populations reported in Yin et al. [40] or Anastos et al. [19], or by individual removal of publications from all comparisons.

## Discussion

In this meta-analysis, low BMD and fracture was increasingly associated with HIV/HCV co-infection compared to HIV mono-infected and HIV/HCV uninfected or seronegative individuals, suggesting that HCV contributes to a burden a disease greater than HIV infection alone. Despite heterogeneity present in the analysis, the visual trends of the forest plots are suggestive of a positive clinical effect in the population. These findings confirm that co-infection is a risk factor for adverse bone health outcomes. This meta-analysis also confirmed the association between HIV mono-infection and low BMD or fracture as reported in previous studies [2], [3]. This is the first attempt to synthesize data across publications to estimate the odds of low BMD or risk of fracture amongst HIV/HCV co-infected patients. Despite significant outcomes, the pooled associations of low BMD or risk of fracture among co-infected patients varied across publications, possibly due to differences in demographics of the study populations.

The inclusion of a co-infected state without regard for the estimated duration of HCV infection relative to HIV infection (and thus, the stage of dynamic fibrosis progression) limits our understanding of infection and its role on either outcome. Although a single study [7] reported significantly greater fracture risk among HCV patients compared to HIV mono-infected patients, we were unable to compare HCV alone to explore the relative contributions of either infection to fractures or BMD. Recent cross-sectional studies have suggested that HCV infected patients with hepatic decompensation have lower BMD than HCV-infected patients with healthy liver function [23], [24], [30]. The non-hepatic outcomes of HCV remain unclear, including mechanisms leading to low BMD and fractures associated with hepatic osteodystrophy [5], [6], [7].

Co-infection may have a compounded negative impact on bone strength due to reduced osteoclastic activity and hepatic osteodystrophy leading to increased fractures. This is consistent with findings in this analysis and Lo Re et al. [30], who reported both high odds of low BMD and risk of fracture compared to mono- and uninfected individuals. Unfortunately, no study in this analysis reported low BMD and risk of fracture as independent outcomes. Therefore, no association between low BMD and fracture risk could be estimated. The theories explaining the causal associations between co-infection and negative skeletal outcomes are reasonable, but require further research into clinical and pathological mechanisms for further clarification.

Risk factors and mechanisms that underlie the increased association between co-infection and low BMD or fracture have not been fully explained. It is likely to be multifactorial, representing complex interactions between infection, traditional osteoporotic risk factors, and ART. It is estimated that up to 9% of all HIV mono-infected patients have low BMD, irrespective of treatment modality [5], [54], [55] due to chronic inflammation leading to bone resorption [56]. HIV itself may also have direct effects on osteoclastic activity leading to osteoporosis, with the incidence greatly increased among those on ART [56]. Initiation of ART has been associated with 2–6% decrease in BMD over the first two years on therapy [11]. This magnitude in BMD reduction is similar to the first two years of a woman in early menopause. With increased duration of therapy, however, the BMD stabilizes or even improves thereafter. Large subsets of the HIV infected populations (both mono- and co-infected) were on ART of some variation. Insufficient data in the publications on ART duration precluded stratification or adjustment of this potential confounding variable. It is possible that ART patients may have biased the estimates towards a stronger association.

Comparisons between publications are only valid in so far as the groups were demographically similar in other respects. Several studies have reported the prevalence of low BMD in HIV infected patients compared to controls [3], [16], [50] but few have adjusted for potential confounding variables and heterogeneity of included study groups (e.g. including individuals who acquired HIV through different modes of transmission). No difference of low BMD was estimated between co-infected patients and HIV/HCV uninfected individuals. This may be attributed to the small number of studies included in this analysis of co-infected individuals where the baseline risk of low BMD may have been greater in controls, which lead to conservative estimates.

No association was found when all female populations [19], [40] were excluded. It is unlikely that the low BMD was entirely attributed to accelerated bone loss during the menopausal transition given that the reported mean ages were less than the average menopausal woman [52], [53] in the United States, where the studies were conducted. Inflammation, leading to bone resorption, and higher risks of vitamin D deficiency have also been reported among mono- and co-infected patients [3], [7], [8]. Most publications reported a primarily middle-aged (40–55 years) male, Caucasian population. However, non-black race is a known risk factor for osteoporosis as noted in a recent meta-analysis by Shiau et al [2]. It was found that race modified the relationship between HIV infection and fracture, but co-infection was not reported. Although matching was reported in one case control study [43], some publications reported notable differences between HIV-infected individuals that may affect BMD, such as advancing age, menopausal status (estrogen deficiency), smoking history, and alcohol consumption. It is unclear if these potentially modifiable risk factors or other potentially important variables, such as dietary calcium intake, use of medications inducing bone loss (e.g. glucocorticoids), use of antidepressants (e.g. serotonin re-uptake inhibitors), or level of physical activity influenced the results [11], [32]. Few publications adequately reported important potential confounding variables (e.g. gender; age; BMI; substance and alcohol consumption; sedentary lifestyle; digestive, renal, and endocrine disorders including diabetes; nadir CD4+ cell count; viral load) that could have potentially biased the measures estimated. Unfortunately, we were unable to adequately adjust in the analysis for this limitation due to inadequate reporting.

Osteopenia is not as sensitive as osteoporosis as a designation of reduced BMD [9]. Developed for post-menopausal women, the T-score has been applied to other adult populations despite controversy around its utility as a metric to quantify bone loss in men and pre-menopausal women [9], [48], [49]. Thus, age and T-scores are the key predictive factors in determining the BMD testing for screening purposes. The utility of estimating the association of low BMD in co-infected patients relates to the risk of future pathologic fracture. There is a continuous non-linear relationship between BMD and fracture risk [8], [46], and measurement of BMD is regarded as the single best predictor of fractures [46], [47]. The sensitivity of BMD for fracture prediction is low over most reasonable assumptions, but the specificity is high. Thus, many fractures will occur in individuals with BMD values in the normal range, but fracture risk is quite low. By contrast, fracture risk is very high in individuals with low BMD.

Reporting of fracture diagnosis varied among publications. Studies comparing results of questionnaires and information obtained by medical charts or ICD databases have been inconsistent with both over-reporting and underreporting [51]. The level of misclassification of incident fractures could not be estimated in the included publications and is a limitation of the present study. However, diagnosis of fractures from ICD databases, insurance claims, and charted data for fractures has been reported to be highly valid [45].

This study had several limitations. First, the potential biases of the original studies, methodological issues, and different strategies for adjusting for confounders could affect the results of this meta-analysis. The cross-sectional association may have been confounded by other unadjusted factors or selection bias. Selection bias may also exist in studies using data from electronic records, claims-based, or primary care databases. Second, the number of cohort studies available was limited and the follow-up durations may not be sufficiently long to be able to detect associations. Third, different publications reported different definitions of incident fracture events that would affect the estimates of prevalence/incidence. However, all reported that pathologic fractures were likely due to low BMD [7], [28], [29], [30], [40], [41], [43] or lead to limited activity post-fracture possibly due to osteoporosis [39]. These differences could have also contributed to the observed high heterogeneity seen within some studies [57], [58], [59]. Last, publication bias may be of concern because studies that report statistically significant results are more likely to be published than studies reporting non-significant results, and this could have distorted the findings in this meta-analysis. Although the Egger’s test and Begg’s test indicated limited evidence of publication bias, the estimation may not be accurate enough as the number of studies may be insufficient.

The findings in this study add evidence to the importance of monitoring for and informing co-infected patients on the negative outcomes affecting the skeletal system. Increasing studies of multiple morbidities are necessary, as the incidence of co-infection with HIV and viral hepatitis increases and patients live longer. Outcomes will also directly benefit patient health through improved care guidelines (e.g. recommendations to improve physical activity to reduce bone loss once infected). Although the clinical validity of *a priori* chosen measures of association (relative effects) compared to absolute effects (important for interpretation) should be considered, the majority of publications reported adjusted odds or rates with insufficient data to estimate absolute risks. Thus, consideration should be made when interpreting the data as doubling of risk (i.e. 100% greater risk) may only mean an absolute difference of 1% (i.e. from 1 to 2% increase in risk).

In conclusion, this meta-analysis suggests that HIV/HCV co-infection is associated with significantly increased association between HIV/HCV co-infection and low BMD and fractures compared to an uninfected or HIV mono-infected population. The estimated significant cross-sectional and longitudinal associations in the analysis could suggest that there may be an increased risk for adverse bone health outcomes, including pathologic fractures, among co-infected patients. However, the impact of other factors on the estimated outcomes, such as HIV or HCV disease severity and duration, could not be determined. Further controlled, longitudinal studies are necessary to clarify the causal nature of HIV/HCV co-infection according to severity of disease, ART (type or duration), and demographic differences on reduced BMD and risk of fracture. This is especially warranted as the risk of skeletal disease is expected to increase in the future as both HIV and HCV-infected population continue to age. Better understanding of this will provide insight and improvement in screening and early treatment of targeted populations to mitigate fracture risk among aging HIV-infected patients.

## Supporting Information

Checklist S1 **PRISMA checklist.**
(DOC)
